# Autophagy as a modulator of cell death machinery

**DOI:** 10.1038/s41419-020-2724-5

**Published:** 2020-07-08

**Authors:** Masayuki Noguchi, Noriyuki Hirata, Tsutomu Tanaka, Futoshi Suizu, Hiroshi Nakajima, John A. Chiorini

**Affiliations:** 1 Division of Cancer Biology, Institute for Genetic Medicine, Hokkaido University, Sapporo, Japan; 2 National Institute of Dental and Craniofacial Research, National Institutes of Health, Bethesda, MD USA; 3 Department of Allergy and Clinical Immunology, Graduate School of Medicine, Chiba University, Chiba, Japan

**Keywords:** Kinases, Macroautophagy

## Abstract

The balance between cell death and survival is a critical parameter in the regulation of cells and the maintenance of homeostasis in vivo. Three major mechanisms for cell death have been identified in mammalian cells: apoptosis (type I), autophagic cell death (type II), and necrosis (type III). These three mechanisms have been suggested to engage in cross talk with each other. Among them, autophagy was originally characterized as a cell survival mechanism for amino acid recycling during starvation. Whether autophagy functions primarily in cell survival or cell death is a critical question yet to be answered. Here, we present a comprehensive review of the cell death-related events that take place during autophagy and their underlying mechanisms in cancer and autoimmune disease development.

## Facts

- Autophagy is a conserved catabolic process essential for homeostasis, however, its role as a protective mechanism in cancer remains controversial.
- The complex cross talk between apoptosis, autophagic cell death, and necrosis underlies the pathogenesis of multiple diseases.
- The serine/threonine kinase Akt and its downstream signaling molecules at the lysosome may have a role in the interaction between the autophagic and apoptotic pathways.
- Autophagy may underly the pathological conditions of cancer and autoimmune diseases by altering metabolic conditions.

## Open questions

1. Whether and how autophagy has a role in the cell death machinery remain unclear.
2. How the three forms of cell death, autophagy, apoptosis, and necrosis are mechanistically interconnected each other remain unclear.
3. How autophagy could underlie the pathogenesis of cancer and/or the carcinogenesis needs to be clarified.
4. How autophagy mechanistically is involved in the pathogenesis of autoimmune diseases needs to be clarified.
5. Understanding the precise modulation of autophagy may lead to novel therapeutic approaches for several diseases, including cancer and autoimmune diseases.

Autophagy is considered to be a conserved survival process prompted by several cellular stress conditions, which prevents cell damage, promotes cell survival in the event of nutrient scarcity, and responds to cytotoxic stimuli. Thus, autophagy serves as a regulator of homeostasis, effectively preserving the balance between the production of cellular components, and the breakdown of damaged or unnecessary organelles and other cellular constituents^1–4^. Although autophagy was originally characterized as a cytoprotective process in yeast under starvation conditions, it is now believed to be a form of cell death in mammalian cells^5–7^ along with the two other major cell death mechanisms—apoptosis and necrosis^8^.

### Roles of lysosome and induction of autophagy

The molecular events associated with apoptosis, including caspase activation, chromatin condensation, DNA cleavage, and plasma membrane degeneration, are well characterized^8–10^. In contrast, the molecular processes that occur between the lysosomal degradation of cytosolic components and autophagic cell death are poorly understood^11^. Following engulfment of endocytosed and autophagocytosed material into the lysosomal/autolysosomal compartment, lysosomal hydrolases participate in the digestion of endocytosed and autophagocytosed material during acute cell death and cancer progression. The increased expression and altered trafficking of lysosomal enzymes participate in tissue invasion, angiogenesis and sensitization to the lysosomal death pathway^12^. Furthermore, the biological roles of lysosomes in specific cellular processes, including nutrient sensing, signaling, and metabolism have been established^4,13^. Lysosomes contain over 20 hydrolytic enzymes that become activated in the acidic lysosomal compartment^6^. During the later stages of autophagy (i.e., macroautophagy) induction, lysosomal acidification is important for maintaining the hydrolytic activity of lysosomal enzymes, such as cathepsin D. Recent studies suggest that the Akt-mammalian target of rapamycin complex (mTORC1) pathway regulates the proton pump [vacuolar H^(+)^-adenosine triphosphatase ATPase (v-ATPase)] in the lysosomal membrane, which transports protons from the cytosol, thereby regulating the lysosomal acidification process required for autophagy induction^1–3,14,15^. In addition, lysosomal interactions between the vaccinia-related kinase 2 (VRK2)-Akt complexes also regulates lysosomal acidification, and subsequent enzymatic hydrolytic activities^16^. Notably, lysosomal membrane permeabilization leads to hydrolytic activation and release of proteolytic enzymes into the cytosol, resulting in cell death—a process often referred to as lysosomal cell death^17,18^, which may differ from autophagy. In this process, lysosomal hydrolytic enzymes and their activation by the acidified state may have a role under various cellar conditions in human diseases^1–3,19^.

### Cross talk between autophagy and apoptosis

Although autophagy was originally described in yeast, it is now being recognized as a mammalian cell survival/prosurvival mechanism. Research has specifically focused on the cross talk that occurs between autophagy and apoptosis as a means to understand how these two types of cell death are interconnected^5,20–23^. Inhibition of macroautophagy following treatment with small interfering RNAs (targeting autophagy-related protein (Atg)5, Atg6/Beclin-1, Atg10, or Atg12), or via pharmacological agents (3-methyladenine, hydroxychloroquine, bafilomycin A1, or monensin), enhances apoptosis-induced cell death as a consequence of reduced mitochondrial membrane stabilization or caspase inhibition^24^. Autophagy-related proteins (Atg)s have been described as regulators of apoptosis. For example, calpain-mediated cleavage of Atg5,which is known to induce autophagy, switches autophagy to apoptosis^25^.

Similarly, Atg12 is a positive mediator of mitochondrial apoptosis, it regulates the apoptotic pathway by binding and inactivating prosurvival B cell lymphoma (Bcl)-2 family members, including Bcl-2 and Mcl-1^25^. Moreover, in the absence of Atg12-Atg3 complex formation, an expansion in mitochondrial mass and simultaneous inhibition of mitochondria-mediated cell death is observed^26^. These results support the notion that not only do autophagic and apoptotic pathways converge in mitochondria, but also that mitochondria provide and ideal molecular platform for counter regulation of autophagic versus apoptotic cell death^5,9,27,28^.

In growth factor-dependent Bax/Bak-deficient cells, autophagy is activated after growth factor withdrawal. These cells activate autophagy with progressive atrophy leading to cell death. This observation supports the notion that apoptosis is not essential to limit cell autonomous survival. Autophagy is essential for maintaining cell survival following growth factor withdrawal and can sustain viability^21^. Tumor necrosis factor (TNF)-related apoptosis and autophagy has been shown to cross talk in various physiological settings^28–30^. Blocking the p62(sequestosome1, SQSTM1)-dependent recruitment of the necrosome to participate in autophagy can activate apoptosis supporting that the machinery of autophagy can control programed cell death^31,32^.

**Fig. 1:**
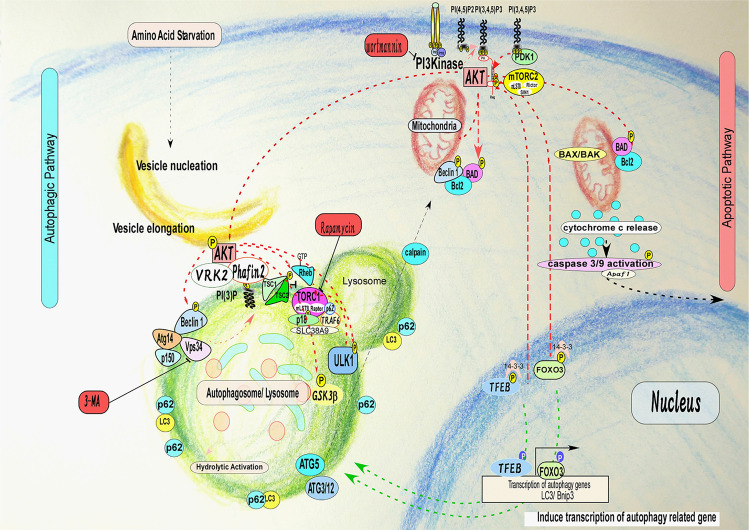
Cross talk regulation between autophagy and apoptosis. Autophagy may contribute to enhanced cell death induced by apoptosis, depending on the relative potency of the cell death-inducing stimuli. It is also possible that apoptosis, autophagy, and necrosis, individually or conjointly, act to induce cell death^8,9,35^. This complex cross talk underlies the pathogenesis and manifestation of cancer likely through alteration of metabolic status via autophagy^9,33,35^. Although autophagy acts as a cytoprotective effect under starvation and/or stress conditions, it is now thought that autophagy also regulates metabolic conditions, which could differentially contribute to the various human diseases such as cancer and autoimmune diseases.

A critical question that remains to be answered is whether autophagy serves primarily as a cell survival mechanism, or rather as an active killing mechanism in mammalian cells^12,29,30^ (Fig. 1). If autophagy is solely a cell survival mechanism, it can protect against cell death induced by either apoptosis or necrosis. However, depending on the relative strength of the cell survival signal elicited through autophagy, cells can either survive or die with a combination of morphological changes associated with autophagic, apoptotic-, or necrotic- cell death. It is possible that depending on the circumstances, typical morphological features of cell death represent those of apoptosis, autophagy, or even necrosis. In contrast what happens in autophagy, the molecular regulation of the cell death cascade is well defined in apoptotic pathways from the death receptor to its downstream signal^31^. However, it remains unclear how lysosomal degradation of intracellular proteins can unavoidably induce cell death^12,20,33^.

There is accumulating evidence regarding the deep connection between apoptosis regulation and the induction of autophagy^7,9,17,18,29,34^, and this cross talk underlies the pathogenesis of, for instance, cancer and its metabolism^9,33,35^. Autophagy is primarily a survival mechanism that maintains the balance between the creation of cellular components and the breakdown of damaged or unnecessary organelles and other cellular constituents. However, autophagy is also known to kill cells under certain conditions, in a process called autophagic cell death, which involves pathways and mediators that differ from those of apoptosis^5,6,23^. It is, therefore, plausible that autophagy enhances cell death caused by apoptosis; alternatively, it may also induce cell death in an apoptosis- or necrosis-independent manner. To support this notion, several experiments have shown that in the molecular process of apoptosis^31^, pro-apoptotic signals, such as TNF-related apoptosis-inducing ligand (TRAIL)^36^, TNF^37^, and FADD^38^ induce autophagy. Ectopic expression of Beclin-1 (Atg6) suppresses cell death, whereas reduced Beclin-1 levels induced by siRNA, sensitizes cells to TRAIL-induced cell death^39^.

### Akt and downstream signaling molecules localize at the lysosome

Akt is a serine/threonine kinase and a major downstream effector that regulates diverse cellular processes through PI3K, and has reported anti-apoptotic, cell proliferative, cell cycle, cytoskeletal organization, vesicle trafficking, and glucose transporting properties^23,40,41^. Furthermore, Akt activation can inhibit autophagy induction in mammalian cells^21,42–45^. To support the roles of Akt in autophagy regulation, Sch9 kinase, a putative yeast orthologue of mammalian Akt and possibly S6K1, is suggested to have a role in the regulation of autophagy^46,47^.

Although the mTORC is considered to be a major factor in the control of autophagy induction, Akt activation can inhibit autophagy in mammalian cells^21,42^. In fact, recent studies have reported that various Akt effector molecules, including mTORC1, mTORC2, glycogen synthase kinase 3β (GSK3β), and the tuberous sclerosis complex (TSC) are present at the lysosome, a major locus for executing autophagy^23,48–52^. The level of phosphorylated Akt, and its substrates at the lysosome remained high even after Hanks Balanced Salt Solution (HBSS) treatment (essentially amino acid deprivation) to induce autophagy, which was sufficient to inhibit general intracellular activation of Akt^16^. Characterization of the protein complexes associated with Akt at the lysosomal membrane after induction of autophagy, showed that Akt forms a complex with Phafin2-vaccinia-related kinase 2 (VRK2), and this complex is important for determining the fate of autophagy in mammalian cells^16,48^.

Akt phosphorylates and inhibits TSC1/2, which leads to the stabilization of Rheb GTPase^40,53^. This in turn activates mTORC1, resulting in inhibition of autophagy^40^. Akt also directly phosphorylates ULK1 (ATG1) and Beclin-1, which control autophagy^23,54,55^. In Myr-Akt-expressing cells, in which Akt is constitutively activated, Beclin-1 is phosphorylated at Ser234 and Ser295 in the presence of Torin 1, an inhibitor of mTORC1, suggesting that Akt controls autophagy in a manner independent of mTORC1^55^. The observation suggests that Akt signaling may be mechanistically linked to autophagy inhibition and tumorigenesis through regulation of the Beclin-1 complex^56^, possibly independent of mTOR^55^.

The involvement of Akt in regulating autophagy is also supported by the fact that Akt directly phosphorylates a wide variety of autophagy regulatory molecules localized at either mitochondria or autophagosomes, including ULK1 (Atg1) at Ser774 through insulin signaling^54^, as well as Beclin-1, and TSC2^23,49^. Akt can also phosphorylate the anti-apoptotic molecule, Bcl-associated death (BAD), at mitochondria^57^, which then releases activated forms of Bcl-2 at the outer membrane to prevent subsequent cytochrome *c* release for triggering downstream caspase activation^56^.

In addition, transcription factor EB (TFEB), a transcriptional regulator of autophagy, is targeted by Akt to inhibit autophagy induction independent of mTORC1^19,58^. These observations provide further support for the involvement of Akt in the autophagy process^23,54,55^.

A recent study reported that Beclin-1 is a substrate of Akt and can regulate tumorigenesis^55^. Akt-mediated phosphorylation of Beclin-1 inhibits autophagy by forming an autophagy-inhibitory complex composed of Beclin-1(Atg6), 14-3-3 proteins, vimentin, and the intermediate filament complex^55^. Beclin-1 also regulates PtdIns(3)P production in response to growth factor stimulation to control the residency time of growth factor receptors. As a consequence, suppressing Beclin-1 sustains growth factor-stimulated Akt, and extracellular signal-regulated kinase activation^59^. Thus, phosphorylation of Atgs is an additional aspect of autophagy regulation^60^.

### Akt–Phafin2–VRK2 forms a complex at the lysosome

PtdIns(3)P-dependent lysosomal accumulation of the Akt–Phafin2 complex is a critical step in inducing autophagy^48^. Yeast two-hybrid studies revealed an interaction between Akt and Phafin2 (EAPF or PLEKHF2), a lysosomal protein with unique N-terminal pleckstrin homology (PH) and C-terminal FYVE domains^48^. These conserved motifs place Phafin2 in a family of proteins known to induce caspase-independent apoptosis via the lysosomal-mitochondrial pathway^61^. Phafin2 controls receptor trafficking through the endosome^62^. However, the exact physiological roles of Phafin2 at the lysosome during the course of autophagy induction have not been thoroughly investigated. Following autophagy induction, Akt translocates with Phafin2 to the lysosome in a PtdIns(3)P-dependent manner following autophagy induction to form the Akt–Phafin2 complex, a critical step in inducing autophagy via interaction with PtdIns (3)P^48^. In this regard, a PtdIns(3)P-specific RNA aptamer has been identified, and its expression inhibits induction of autophagy as determined by LC3II/I conversion, p62 degradation, formation of LC3 puncta, lysosomal accumulation of Phafin2, lysosomal acidification, and subsequent inhibition of the hydrolytic activity of cathepsin D^63^. Given the roles of autophagy in cancer, infectious diseases, and autoimmune diseases, this RNA aptamer may have possible implications for the design of novel therapeutics^63^.

Through time-of-flight/mass spectrometry analysis of lysosomal Akt protein complexes, VRK2 was found to interact with Akt and to be enriched at lysosomes following autophagy induction^16^. VRK2 interacts with Akt in a kinase-dependent manner to maintain the phosphorylation of Akt at the lysosome. Ectopic expression of the active form of Akt, but not kinase-dead Akt, can induce autophagy induction suggesting the kinase activity of Akt is critical for induction of autophagy.

**Fig. 2:**
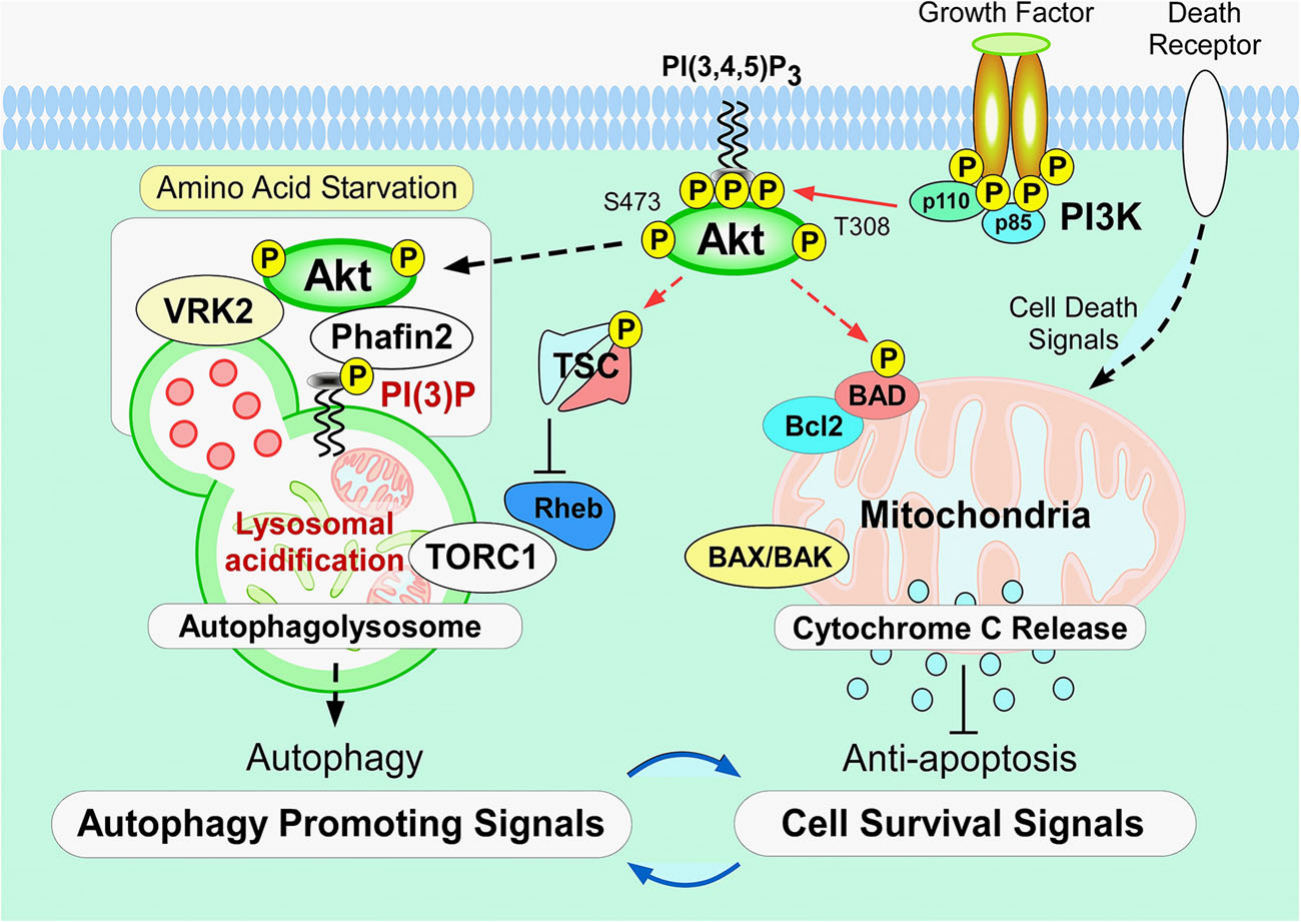
Lysosomal Akt–Phafin2–VRK2 complex as a functional modulator of autophagy. Regulatory roles of Akt at the lysosome in the course of autophagy are suggested by Beclin-1 (Atg6), transcription factor EB (TFEB), and Unc-51 like autophagy activating kinase 1 (ULK1) functioning as direct substrates^23,54,55^. Recent investigations revealed that target molecules of Akt, such as the tuberous sclerosis complex (TSC), Beclin-1 (ATG6), ULK1, and BAD, are present in the lysosomes^23,48–52^. Lysosomes are intracellular organelles that participate in intracellular protein degradation through activating hydrolytic enzymes in the process of autophagy for amino acid recycling^8–10^. Autophagy, a cell survival mechanism during starvation conditions, participates in the regulation of cell death/survival machinery^5–7^. Searching for the binding partner of Akt at the lysosome using yeast two-hybrid screening and time-of-flight/mass spectrometry analyses, we identified the formation of a Akt–Phafin2–VRK2 protein complexes at the lysosome in a phosphatidylinositol 3-phosphate (PtdIns(3)P)-dependent manner^16,48^. A functional study demonstrated the regulatory roles of the Phafin2–VRK2–Akt protein complex at the lysosome in the course of autophagy induction. These findings support the notion that the intersection between autophagic cell death and apoptosis may be at the mitochondria. The presence of the Akt–Phafin2–VRK2 at the lysosome sheds light on the roles of cell death and survival machinery in mammalian cells that may underlie various human diseases including cancer and autoimmune diseases.

VRK2 facilitates lysosomal localization of activated Akt and its substrates after HBSS treatment to induce autophagy. Functional characterization of the VRK2–Akt interaction clarified the roles of VRK2 in sustaining the kinase activity of Akt at the lysosomes, which controls the size, acidification, and induction of autophagy in mammalian cells. Overall, these findings support a model in which spatial control of Akt and its kinase activity toward the lysosome serve as functional modulators of autophagy induction via VRK2A. Moreover, the functional interaction between Akt–Phafin2–VRK2 complex at the lysosome supports the hypothesis that spatial localization of activated Akt at the lysosomes has important roles in the process of autophagy induction, which many contribute to the regulation of autophagic versus apoptotic cell death^16,23^ (Fig. 2).

### Mitochondria at the crossroad of cell death

Mitochondria-associated proteins may also be responsible for the cross talk between the autophagic and the apoptotic pathways^5,9,27,28^. Bcl-2 inhibits Beclin-1-dependent autophagy^64,65^ and phosphorylation of Beclin-1 on T119 via death-associated protein kinases, inhibits the Bcl-2–Beclin-1 interaction and activates autophagy^66^. The protein complex composed of Beclin-1 and Bcl-2, which physically interacts at the mitochondrial outer membrane, is regulated by calpain-mediated cleavage of Atg5, a key regulator of apoptosis^67^. Ayg3/12 and Bim, another member of the Bcl-2 family of proteins, have also been shown to inhibit apoptosis via mitochondria^26^. In this regard, it is worth noting that mitochondria are also targeted for lysosomal-mediated degradation in a process known as mitophagy, a selective degradation of mitochondria by autophagy that often occurs to remove and digest defective mitochondria following damage or stress^68^. Decreased expression of genes that regulate autophagy or mitophagy can cause degenerative diseases in which deficient quality control results in inflammation and the death of cell populations. Thus, the altering of mitochondrial functions is suggested to be associated with multiple degenerative diseases^5^.

Beclin-1 and ULK1 (Atg1) are both involved in autophagosome formation and are direct substrates of Akt^54,55^. Bcl-2 is an anti-apoptotic protein that acts as a major effector of Akt signaling through BAD phosphorylation, which maintains the mitochondrial outer membrane potential to modulate cell survival^69–71^, in part by inhibiting Beclin-1-dependent autophagy^64,71,72^. Therefore, it is possible that mTORC1 (via TRAF6-p62-mediated ubiquitination)^73^ and Akt (via Phafin2–VRK2-induced autophagy) serve as molecular links between autophagy and apoptosis^23,48^.

### Autophagic cell death

Three mechanisms for cell death are reported in mammalian cells; apoptosis (type I), autophagic cell death (type II), and necrosis (type III)^6,74^. To date, it is not entirely clear whether autophagy is indeed a cell survival mechanism or rather a cell death-inducing mechanism in mammalian cells^8,27,75^. Autophagy is believed to be a conserved mechanism, in diverged organisms, for the gross disposal and recycling of targeted intracellular proteins^13,43,76^. However, it also induces cell death with morphological findings that differ from those of apoptosis or necrosis; referred to as “autophagic cell death” or “type II cell death”^8,74,75,77^.

Apoptotic cell death (type I cell death) is characterized by morphologic changes including plasma membrane blebbing, apoptotic bodies, and nuclear condensation with reduction of cell volume^7^. Alternatively, necrotic cell death (type III cell death) is characterized by a gain of cell volume or swelling of organelles often associated with plasma membrane rupture without blebbing and subsequent loss of intracellular content. Notably, necrosis can occur as a consequence of apoptosis^7,74^. Autophagic cell death (type II cell death) is characterized by the appearance of cellular vacuoles without extensive condensation of the nucleus or caspase activation^7,74^. Morphologically, autophagic cell death differs considerably from cells undergoing apoptosis or necrosis, and is characterized as a form of cell death that mechanistically depends on the autophagic machinery with extensive cytoplasmic vacuolization, phagocytic uptake, and lysosomal degradation^78^.

Lysosomes can initiate a cell death pathway, designated lysosomal cell death, which is distinct from autophagic cell death^8^. Lysosomal cell death is mediated by the release of cathepsins into the cytoplasm following lysosomal damage^79^. However, the molecular processes that occur between the lysosomal degradation of cytosolic components and autophagic cell death are not well characterized^11^. It is also possible that the three biological mechanisms of cell death (apoptosis, autophagic cell death, and necrosis) act independently or interactively cross regulate each other in cell death and survival machinery^8,9,35^.

Inactivation of lysosomal membrane-associated protein (LAMP)2 by RNA interference or by homologous recombination leads to autophagic vacuolization in nutrient-depleted cells. Furthermore, cells that lack LAMP2 expression showed an enhanced accumulation of vacuoles carrying the marker LC3, yet a decreased colocalization of LC3 and lysosomes. Thus, supporting the complex relationship between localization of LC3 at the lysosome and enhanced vacuole formation in the course of autophagic cell death and apoptosis^80^.

**Fig. 3:**
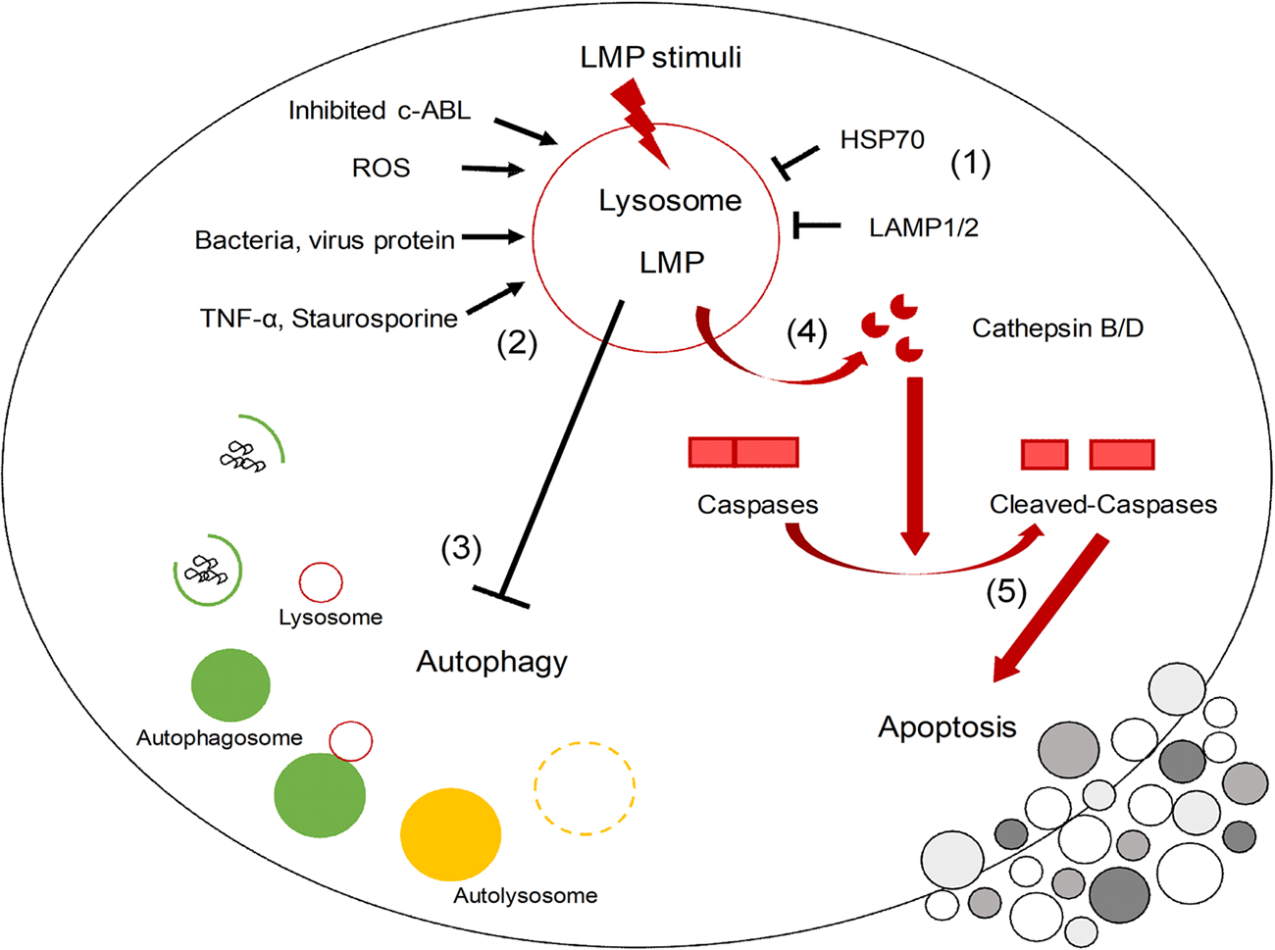
The lysosome and lysosomal membrane permeability are central to the balance of autophagy and apoptosis. (1) LAMP1, LAMP2, and HSP70 protect lysosomal membrane integrity from lysosomal membrane permeability (LMP) stimuli. (2) Lack of these protein, or increased LMP stimuli such as reactive oxide species (ROS), c-ABL dysfunction, bacteria or virus infection, TNF-α, and staurosporine treatment can disrupt lysosomal membrane integrity resulting in LMP. (3) The decrease in matured lysosomes induced by LMP leads to inhibition of autophagy-dependent protein degradation. (4) LMP also initiates relocalization of lysosomal enzyme such as cathepsins into cytoplasm. (5) Released cathepsins activate caspases resulting in apoptosis.

Recent papers further indicate that LAMP3 has a role in autophagy^81,82^. In these studies, LAMP3 knockdown cells exhibit a suppressed ability to complete the autophagic process. Furthermore, although death by lysosomal membrane permeabilization (LMP) and subsequent release of cathepsins into the cytoplasm has been previously reported, the mechanism of action is not well characterized and the inducing agents have only been described as compounds capable of sensitizing cancer cells^18,83^ (Fig. 3). Furthermore, LMP can induce cell death via several mechanisms including both caspase-dependent and independent pathways, as well as via activation of the inflammasome to release cytokines^84^. A common characteristic of LMP-associated cell death is sensitivity to cathepsin inhibitors such as pepstatin A^85^. In fact, cytoplasmic cathepsins are reported to cleave Bid and induce translocation of the pro-apoptotic proteins Bax and Bak, which in turn induce mitochondrial membrane permeabilization and caspase-dependent apoptosis^86^.

### Autophagy as an underlying mechanisms of cancer

Apoptotic cell death and its evasion are shown to be directly involved in pathogenetic conditions of human cancer^87–90^. For example, activation of Bcl-2, an anti-apoptotic factor, via chromosomal translocation serves to enhance cell survival, which contributes to development of human B cell lymphoma. Furthermore, we previously reported that TCL1 functions as an Akt coactivator by enhancing transphosphorylation within the TCL1-Akt high molecular weight protein complex to enhance cell survival, thereby serving as an underlying molecular mechanism of human T cell prolymphocytic leukemia^91–94^. Alternatively, the tumor-suppressor gene *PTEN* dephosphorylates phosphatidylinositol (3,4,5) triphosphate (PIP3) and phosphatildylinositol (3,4) bisphosphate to inhibit Akt activation^95,96^. Loss of PTEN function leads to an elevated concentration of the PIP3 substrate, and consequent constitutive activation of the downstream components of the PI3K pathway including Akt and its downstream effector mTOR, underlying development of human glioblastoma, melanoma, prostate cancers, lung cancers, and lymphomas^97,98^. Consistently, PTEN knock-out mice revealed a high incidence of neoplastic diseases including T cell lymphomas, gonadostromal tumor, germline cancers, and, thyroid, prostate, breast, liver, and intestinal tract cancers^96,99^.

Metabolic regulations are important for controlling cell death^27,100^. The PI3K–Akt signaling pathways^40,95,97,101^, originally characterized to mediate insulin signaling, are critical metabolic pathways in mammalian cells required to divert nutrients toward anabolic processes to facilitate enhanced growth and proliferation^102–104^. Altering the metabolic state in cancer cells, which was originally described as the Warburg effect, increases aerobic glycolysis and is considered to be metabolic hallmarks of cancer cells through both post-translational and transcriptional regulation^105,106^. The PI3K–Akt–mTOR pathway coordinates the uptake and utilization of multiple nutrients, including glucose, glutamine, nucleotides, and lipids, in a manner best suited for supporting the enhanced growth and proliferation of cancer cells^107,108^.

Autophagy degrades and recycles proteins and organelles to support metabolism and survival in starvation. Given the possible roles of autophagy in the regulation of cell death machinery, it is possible that it may underlie molecular mechanisms of altered metabolism in human cancer^100,109,110^. Indeed, autophagy can act as a suppressor of early tumorigenesis through controlling cancer metabolism^27,109^.

Autophagy was initially considered a tumor suppressive mechanism based on indirect evidence from oncogene and tumor-suppressor gene alteration studies^100^. In addition autophagy can support tumor growth in multiple tumor types^111,112^, as well as promote resistance to a variety of therapies^113,114^. Amplification, or gain of function mutations in *PI3K*, *AKT*, or loss of function *PTEN* mutations, serve to activate mTOR thus inhibiting autophagy, and have, therefore, been defined as underlying mechanisms for oncogenicity, suggesting a potential importance of suppressing autophagy during tumor initiation^112,115^. To support this notion mice lacking the *atg7* gene were created concurrently with *K-ras*^*G12D*^ activation to investigate non-small-cell lung cancer. Results found that *Atg7*-deficient tumors accumulated dysfunctional mitochondria and prematurely induced p53 and proliferative arrest, which reduced tumor burden that was partly relieved by p53 deletion^116^. In some contexts, autophagy suppresses tumorigenesis, however, in most contexts autophagy facilitates tumorigenesis^100,111^. Autophagy is shown to promote tumor growth by suppressing the p53 response, maintaining mitochondrial function, sustaining metabolic homeostasis and survival in stress, and preventing diversion of tumor progression to benign oncocytomas suggesting that cancers require autophagy for distinct roles in metabolism that are oncogene- and tumor-suppressor gene-specific^111^. In this scenario, cancer cells may upregulate autophagy to survive microenvironmental stress and to increase growth and aggressiveness^100,111^. The signaling link between p62 (sequestosome1, SQSTM1), a core autophagy regulator, and the apoptosis pathway was suggested as a critical decision point that controls cell death and survival in tumorigenesis^32^. Mice with systemic mosaic deletion of *Atg5* and liver-specific *Atg7*(−)/(−) mice develop benign liver adenomas with mitochondrial swelling, p62 accumulation, and oxidative stress and genomic damage responses. The size of the *Atg7*(−)/(−) liver tumors was reduced by simultaneous deletion of p62^117^. These observations together suggest that, analogous to the role of autophagy in normal tissues, that in tumor tissues appear to be complex and context-dependent^9,100,109,118^.

In addition to the fundamental roles of autophagy as a lysosomal degradation pathway for nutrient/amino acid recycling, autophagy can mediate various autophagy-independent functions, such as metabolic adaptation, cell proliferation, exosome secretion, pathogen control, centrosome functions, endocytosis, phagocytosis, inflammation, and innate immunity^11,74^. Thus, the involvement of autophagy in cancer pathogenesis may not be solely through controlling cell death machinery, but also through alteration of various cellular conditions. Therefore, attention has turned to autophagy as a pathological survival-promoting pathway for cancer metabolism^5,6,27,28,33,75,100^ underlying the mechanisms responsible for initiation and maintenance of various types of cancers^55,100,117,119–121^. Mechanisms by which autophagy promotes cancer include suppressing induction of the p53 tumor-suppressor protein and maintaining metabolic function of mitochondria^100^. Autophagy can impede early cancer development while facilitating advanced tumor progression^33,109^. Autophagy can impede early cancer development while facilitating advanced tumor progression^33,109^. Indeed, autophagy is suggested to underlie the conditions of various aspect of cancer and its pathological conditions including alteration of metabolic condition^33,109^.

A primary cilium is a microtubule-based sensory organelle that has an important role in human development and disease^122^. Loss of cilia has been observed in multiple malignant tumors, suggesting a potential suppressive role of cilia in cancer development through the dual interaction between autophagy and ciliogenesis^123,124^. Akt, primary regulator of cancer development, physically interacts with Inversin at the primary cilia controls normal cilial development^125^. Therefore, it is possible that Akt may have a regulatory role through the interplay between cilia and autophagy to alter metabolic status for cancer development.

Furthermore, chaperon-mediated autophagy (CMA), the direct delivery of cytosolic proteins targeted for degradation to the lysosome^126^, is suggested to exert a major regulatory effect on cell metabolism^127^. Therefore, it is possible that autophagy-induced alteration of energy homeostasis and cell metabolism may underlie the manifestation of cell death associated with cancer. Thus, autophagy inhibition can limit the growth of established tumors to potentially improve responses to cancer therapeutics^114^.

### Autophagy and autoimmune diseases

Dysregulation of autophagy has been implicated in a variety of autoimmune diseases. Several lines of evidence from animal models, genome‐wide association studies (GWAS), and human patients are emerging to support the role of autophagy in progression and pathogenesis of systemic lupus erythematosus (SLE), a prototypic autoimmune disease characterized by the production of multiple autoantibodies in association with systemic inflammation and organ damage^128^. GWAS have identified several autophagy‐related genes (*ATG5*, *ATG16L2, CDKN1B*, *CLEC16A*, and *DRAM1*) that are associated with susceptibility to SLE (reviewed in ref. ^129^). In addition, several variants located in other autophagy‐related genes including *ATG7*, *APOL1*, *IRGM*, *LRRK2*, *MAP1LC3B*, and *MTMR3* are associated with susceptibility to SLE^129^. This study strongly supports the notion that autophagy has an important role in the genetic etiology of SLE.

**Fig. 4:**
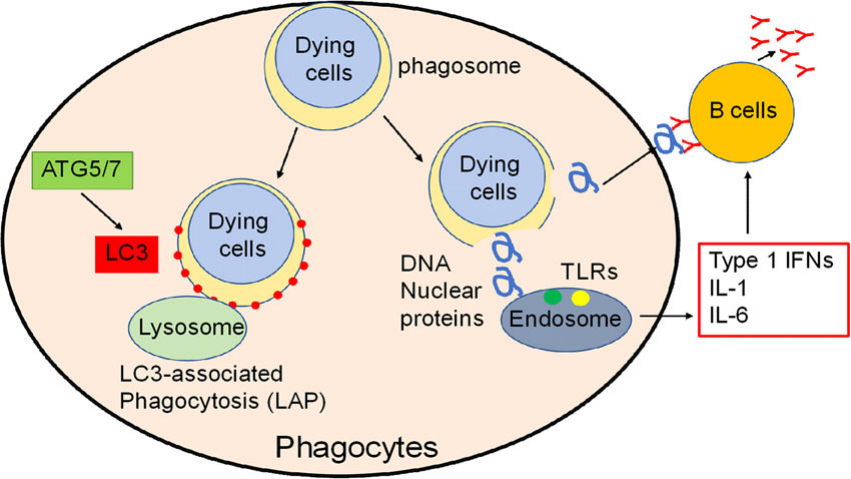
LC3-associated phagocytosis (LAP) in the clearance of dying cells. Generally, phagosomes become decorated with LC3 and are then trafficked to the lysosome for degradation (LC3-associated phagocytosis (LAP)). LAP uses a subset of the autophagy machinery distinct from canonical autophagy. As a result, dying cells are rapidly ingested and degraded and do not induce an autoimmune response. If components of the LAP pathway are lacking, dying cells are not degraded and, hence, the nuclear material escapes, which leads to autoimmune responses. Consequently, a lupus-like systemic inflammatory disease develops.

In the pathogenesis of SLE, immune complexes of multiple autoantibodies with nuclear autoantigens can deposit in various tissues and stimulate cytokine production to cause inflammation^130^. The extracellular release of nuclear autoantigens is likely to be a critical step in the pathway. Although the release is considered to be the consequence of apoptotic cell death, recent findings show that other forms of cell death, including autophagy may be similarly involved. Indeed, enhanced autophagy has been reported in T and B cells from a lupus mouse model, as well as in PBMCs from patients with SLE^131^. In addition, autophagy-related genes, including mTOR, Beclin-1 (Atg6), LC3, and p62, have been shown to be differentially expressed in PBMCs in SLE patients^132^. Importantly, mice lacking components of the LC3-associated phagocytosis (LAP) pathway show increased serum levels of inflammatory cytokines and autoantibodies, as well as kidney damage with glomerular immune complex deposition^133^. Moreover, repeated injection of dying cells into LAP-deficient mice accelerates the development of SLE-like disease^133^, suggesting the defects in LAP rather than canonical autophagy, may contribute to the pathogenesis of SLE (Fig. 4).

**Fig. 5:**
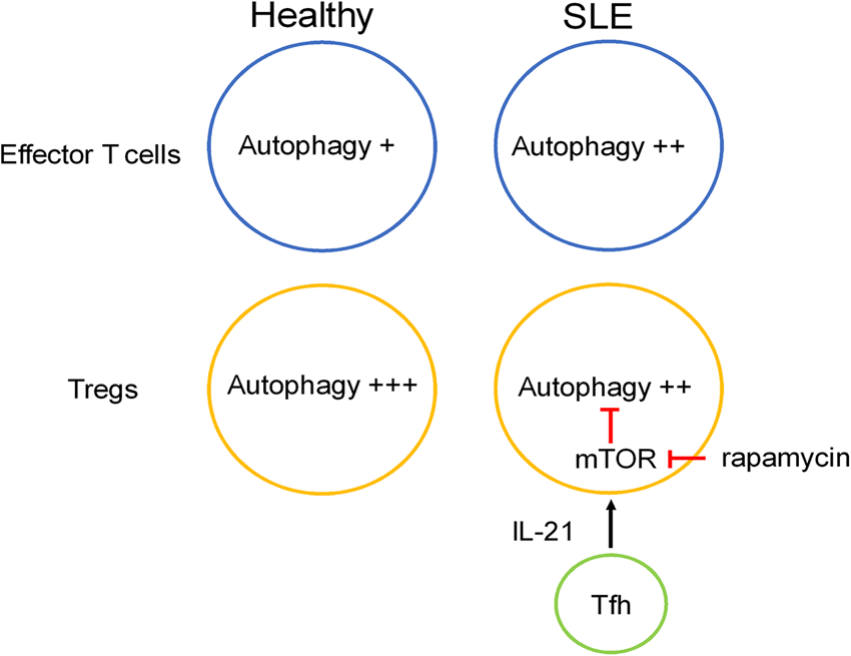
IL-21-mTOR axis in the regulation of autophagy in Tregs in SLE. Autophagy is profoundly diminished in Tregs in SLE. IL-21 induces mTOR activation and eliminates autophagy in Tregs. The blockade of mTOR by rapamycin induces autophagy and recovers the function of Tregs.

Dysfunction of the immune systems, including B cells and T cells of the adaptive immune systems as well as macrophages, dendritic cells (DCs), innate lymphoid cells (ILCs), and neutrophils of the innate immune systems, are considered to be involved in the pathogenesis of SLE^130^. Among them, dysfunction of regulatory T cells (Tregs) is likely to be deeply involved^134^. Indeed, in a murine model of SLE, the number of Tregs decreased along with disease progression^135^. In addition, the suppressive function of Tregs decreased in patients with active SLE^136^. Consistent with an earlier mouse study by Wei et al.^137^, autophagy has been shown to be augmented in Tregs over effector T cells in healthy controls^134^. However, the difference in autophagy is abolished in SLE patients as autophagy is enhanced in naive and proinflammatory effector CD4^+^ T cells yet profoundly diminished in Tregs in SLE^138^. Importantly, IL-21, a key cytokine produced by follicular helper T cells, induces mTOR activation and eliminates autophagy and differentiation of Tregs^138^ (Fig. 5). Consistently, dual blockade of mTORC1 and mTORC2 by rapamycin treatment induces autophagy, which restores the expression of GATA-3 and CTLA-4, and recovers the function of Tregs^138^. These findings suggest that increased activity of the IL-21–mTORC1 axis in SLE results in the blockade of autophagy in Tregs, leading to the development of SLE. Consistently, rapamycin has been shown to be efficient in treating SLE patients as well as lupus mice^139^.

Recent reports also suggest a role for autophagy in Sjögren’s syndrome, a complex autoimmune disease of secretory glands. Markers of macroautophagy are reported to be increased in tears, conjunctival cells, and salivary glands of primary Sjögren’s patients^140,141^. Investigation in MRL/lpr mice, which spontaneously develop systemic autoimmune disease that is similar to human SLE and secondary Sjögren’s syndrome, showed that markers of macroautophagy flux and CMA are reduced^142^. Although these findings seem contradictory, given the role of autophagy in protein degradation and release, markers of autophagy may be expected in gland secretions if the natural progression of protein degradation is affected. Additional studies of the minor salivary glands of Sjögren’s syndrome patients suggest that LAMP3 is upregulated in a subset of patients and contributes to a stalling of autophagy and an increase in LMP. This increase in LAMP3 also results in the release of autoantigens via a caspase-independent process, which may account for the release of other marker of autophagy by exocrine cells. Further studies will be required to more accurately describe whether the role of autophagy is central to the development of Sjögren’s syndrome or if this is a byproduct of cellular dysregulation.

In addition to SLE and Sjögren’s syndrome, the combined data from GWAS and analyses in murine models have implicated autophagy in other autoimmune diseases, including rheumatoid arthritis (RA), multiple sclerosis (MS), and Crohn’s disease. RA is an inflammatory autoimmune disease that primarily affects the joints. Although the role of autophagy in the survival of synovial fibroblasts remains controversial, increased levels of autophagy have been shown in the synovial tissues of active RA patients^143^. MS is an inflammatory disorder that is characterized by immune system reactivity against myelin in the central nervous system, resulting in relapsing or progressive neurological degeneration. Inhibition of autophagy in CD4^+^ T cells has been shown to exhibit a protective role in experimental autoimmune encephalomyelitis (EAE)^144^, a murine model of MS. Consistently, rapamycin treatment reduces the disease activity of EAE via inhibition of autophagy^145^. Moreover, GWAS have identified Atg16L1 and immunity-related GTPase family M (IRGM) in Crohn’s disease^146^, indicating a role of autophagy in the pathogenesis of Crohn’s disease.

### Conclusion and perspective

Autophagy, originally described as a cellular survival mechanism initiated upon starvation conditions, underlies various aspect of cellular events by recycling intracellular proteins at the lysosome^1–4^. However, under certain conditions such as cancer pathogenesis, autophagy can induce cell death both directly and indirectly^5–7^. Experimental approaches including targeted deletion *Atg* genes in mice^111^, support a biphasic complex role for autophagy in the context of cell death^109^. Among the wide variety of cellular responses controlled by autophagy, metabolic function may have a key role in the progression of autophagic cell death^8^. Clarification of the roles played by autophagy in the cell death machinery will open a new avenue for the treatment of disease in which autophagy modulation may be a novel and impactful molecular target for cancer therapy and autoimmune diseases^7,147^.
